# A Standardized Reporting System for Assessment of Diverse Public Health Programs

**DOI:** 10.5888/pcd9.120004

**Published:** 2012-09-13

**Authors:** Douglas Fernald, Abigail Harris, Elizabeth Ann Deaton, Vicki Weister, Shannon Pray, Carsten Baumann, Arnold Levinson

**Affiliations:** Author Affiliations: Abigail Harris, Vicki Weister, Shannon Pray, Arnold Levinson, University of Colorado Denver, Aurora, Colorado; Elizabeth Ann Deaton, University of Denver, Denver, Colorado; Carsten Baumann, Colorado Department of Public Health and Environment, Denver, Colorado.

## Abstract

State public health agencies face challenges when monitoring the efforts and effects of public health programs that use disparate strategies and address various diseases, locations, and populations. The external evaluators of a complex portfolio of grant funding sought a standardized reporting framework and tool that could be used for all grants in the portfolio, without having to redesign it for each disease or intervention approach. Evaluators iteratively reviewed grant-funded projects to identify common project delivery strategies, then developed and implemented a common reporting framework and spreadsheet-based data capture tool. Evaluators provided training, technical assistance, and ongoing data reviews. During 2 fiscal years, 103 public health programs throughout Colorado submitted quarterly reports; agencies funded to implement these programs ranged from small community-based organizations to university- and hospital-affiliated groups in urban and rural settings. Aggregated reports supported estimates of program reach by strategy and by disease area, and the system supported production of summary descriptions of program implementation. Standardized language and expectations for reporting helped to align grant applications and work plans with reporting tools. A common language and standardized reporting tool can be used for diverse projects in a comprehensive evaluation framework. Decentralized data collection using common spreadsheet software enabled the aggregation of common data elements across multiple programs and projects. Further refinements could enable wider dissemination of common reporting criteria and expectations.

## Introduction

Large public health initiatives are hard to monitor and evaluate when the health objectives involve several diseases, dispersed geography, multiple populations, or comprehensive strategies being delivered by varied public and private entities with diverse evaluation capacity. Even modifying a single project for delivery to multiple sites complicates monitoring and evaluation and is often expensive (1). Previous researchers have suggested strategies for gathering standardized multisite information based on homogeneous programs or interventions focused on a single disease or delivery strategy (2-4). Theoretical frameworks and guides exist for evaluating large, single-objective public health programs and their individual projects (5-12). However, little is known about practical assessment strategies for large, dispersed, and heterogeneous public health program portfolios.

One such portfolio was created in 2004 when Colorado voters approved a tobacco-tax increase and earmarked a portion of revenue streams to address illnesses related to tobacco use through extending programs and translating evidence-based approaches. In the portfolio, 2 grant-making programs address highly diverse public health needs: 1) reduction of health disparities in illnesses related to tobacco use and 2) prevention, early detection, and treatment of cancer and cardiovascular and pulmonary diseases (CCPD). These 2 programs generated 103 unique projects, which were conducted during fiscal years (FYs) 2006 through 2009 with approximately $100 million of tobacco-tax revenues. Agencies funded to implement these programs ranged from small community-based organizations to university- and hospital-affiliated groups in urban and rural settings.

The number and diversity of these projects introduced monitoring and evaluation challenges. At a minimum, the legislation that created the grant-making programs requires annual reporting of each project’s implementation (number of people served, services provided) and overall results of aggregate program activities. Administrative oversight requires capacity to track grantee activities and compare them to initial work plans for accountability. Professional public health practice requires state-level information to guide periodic planning, program refinement, and project continuation decisions. Grantees need formative feedback for quality improvement and summative feedback to reinforce motivation and maintain enthusiasm. Because of the array of data required, an efficient and standardized evaluation system was needed.

## Program and Evaluation Setting

The state department of health (DOH) engaged a university-based evaluation group to develop strategies to meet these monitoring and evaluation needs. The resulting evaluation system includes state-level surveillance of program-related outcomes, a range of special studies, and a standardized approach for collecting, analyzing, and reporting data from all funded projects. We describe the design, implementation, and utility of the standardized project monitoring and evaluation program.

The monitoring and evaluation system portfolio addresses prevention and treatment interventions for multiple diseases, generating data elements that are neither comparable nor compatible in format nor collected in the same way or during similar times. For example, colorectal cancer screening can yield several types of positive findings (eg, polyps, cancer stages), each of which generates different follow-up needs; smoking cessation treatment outcomes include treatment type, duration of use, and abstinence status at follow-up. These 2 examples illustrate the futility of trying to meaningfully standardize results and report aggregate “success.” However, a substantial literature indicates that public health projects should faithfully apply methods known to be effective, and doing so makes outcome evaluation somewhat less urgent (2,12-14). Adopting this perspective, the evaluation team and the DOH decided to rely on a priori funding eligibility criteria that require projects to adopt strategies that have been evaluated as effective. This decision allowed the team to focus evaluation and monitoring aims on 1) detailed description of populations served (*reach*), and 2) assessment and summary of project activities (*implementation*). The DOH felt that meaningful outcome evaluation of every project would have diverted too many resources from program delivery. Instead, outcome evaluation was planned and ultimately conducted for selected projects using focused selection criteria (ie, project size, interest, or need for more detailed information).

### Development of a reach and implementation reporting system

Several principles guided development of a reporting system that would acceptably balance the needs of grantees, the grant-making programs, and the DOH (Box). We worked with DOH staff to develop a system that fulfilled the required quarterly reporting by grantees, avoiding additional reporting burden or duplicate data entry. We first conducted an iterative review of all funded projects to identify common strategies that were being employed. Analysts generated an initial list that was reduced to 14 reporting categories (Table 1), each designated as *direct* (serving community members or patients) or *indirect* (aimed at intermediaries such as health care providers, community health workers, or organizations).

**Box. Guiding Principles for Reporting System Development**

**Table:** 

1. Accept a large number of data elements across a range of project designs, disease areas, populations, scopes, and funding levels.
2. Provide data needed to meet legislative requirements.
3. Align with overall program goals.
4. Support quarterly reporting and contract monitoring.
5. Align with pre-existing project work plans.
6. Define implementation by using a portable, field-ready, decentralized and broadly applicable model.
7. Employ off-the-shelf software rather than create a costly customized system.
8. Obtain quality data without sacrificing user-friendliness.
9. Provide data entry pathways that are easy for grantees with little or no evaluation capacity to follow.
10. Avoid burdening grantees with duplicate data entry or reporting requirements.

**Table 1:** Direct and Indirect Program Delivery Strategies Used in the Reporting System

Strategy type	Sample Activities
**Direct (targeting community members and patients)**	
Access to care	Fee reduction, vouchers, patient transportation services
Awareness/media	Brochures, posters, handouts, presentations, newsletters, print and broadcast media (purchased or earned)
Disease management	Patient navigation, case management, self-management
Education/training	Health education for patients or community members
Referrals	Refer identified individuals to follow-up care or services
Risk factor reduction	Programs for weight loss, physical activity, nutrition, smoking cessation, sun protection
Screening	Screen individuals for specific illnesses or risk factors
Treatment	Provide appropriate condition-specific treatment
**Indirect (targeting intermediaries, including health care providers, community health workers, program staff, or organizations)**	
Awareness/media	Advertising campaigns, handouts, informational meetings
Collaboration/partnership	Build or enhance joint efforts with other agencies, organizations, businesses
Data collection/analysis	Collect or analyze primary or secondary data as a primary grant activity
Education/training	Training clinicians, providers, other health care workers; continuing education
Infrastructure	Acquire equipment, staff/personnel, supplies, skills, systems, other resources
Policy	Develop, ratify, implement policies

We defined category-appropriate metrics to enable coarse-grained assessment of implementation completeness and intensity. The reporting forms prompt training projects to indicate the types of sites where the training took place and to enumerate how many total sites were involved. These projects also report how many training sessions each person participated in and the average length of each session to enable analysis of training duration and intensity. We defined reach metrics using standard demographic items (eg, sex, age group, race/ethnicity) and characteristics reflective of disparities (eg, income level, education level).

Final reporting forms use Microsoft Excel (Microsoft Corp., Edmonds, Washington), providing consistent navigation and information features — drop-down lists, gray-out functions, and information pop-ups. Every project completes a separate worksheet for each strategy employed. Projects update workbooks quarterly and send them to the evaluation team for review, data verification, and compilation. Data cumulate across quarters, eliminating a need for annual project reports and enabling annual evaluation from fourth quarter reports. This report about evaluation processes and tool development involved no research on human subjects and was not subject to review by an institutional review board.

### Reach and implementation system deployment

Grantees receive an initial half-day training session to learn the new reporting system and to affirm mutual values that could be served, which fosters program receptivity among grantees, identifies reduction in reporting burden, and demonstrates sincerity on the part of program creators to obtain critical feedback from grantees. An incremental approach has been recommended for introduction and revision of a standardized reporting system (15). We assured participants that the first year would be devoted to learning the new system and to revising and improving it. We also informed them that subsequent technical assistance would consist of quarterly data reviews and both group and one-on-one conference calls to obtain participant experiences with the system and to address problems.

After pilot-testing with a small sample of grantees that had varying degrees of evaluation capacity, the DOH launched the system during FY 2008–2009. Health disparities and CCPD grantees received the half-day training session, reporting forms, and a user’s guide. The evaluation team reviewed quarterly data submissions and provided grantees with feedback about data quality. The team also generated lists of operational issues, a few of which required immediate solutions but most of which were addressed in a second, streamlined reporting tool that was deployed in FY 2009–2010.

### Data management and analysis

Analysts process quarterly report files aggregating data from each field, organized by project and quarter. Individual project reports are reviewed for missing and out-of-range data and information that cannot be interpreted on the basis of prior knowledge of the project activities. After each of the first 3 quarters, the DOH sent projects a summary of the reviews with requests for corrections or clarifications. Fourth-quarter reports were reviewed, and final requests for corrections were issued to grantees. The evaluation team uses fourth-quarter reports to generate annual program reports with summary tables and narrative description of aggregated reach and implementation.

## Use of the Reports and Data

All grantees (n = 98) completed quarterly reports in the new system during FY 2008–2009 and FY 2009–2010. Most projects used multiple direct and indirect strategies in accordance with their proposals. Various approaches to aggregation and reporting are possible.

### Estimated program reach across projects

One approach is to combine reach data across programs and their respective projects. The total number of people reached in an FY can be reported by demographic characteristics (eg, race/ethnicity, sex, age) or by chronic disease area and strategy (Table 2).

**Table 2:** Estimate of People Reached Statewide and Numbers of Projects, by Disease Area and Strategy, Colorado, Fiscal Year 2008–2009

Strategy	Disease Area				Total (N = 85)^a^
	Cancer (n = 15)	CPD (n = 6)	CVD (n = 29)	Cross-Cutting^b^ (n = 35)	
Access to care	1,069 (n = 6)	0 (n = 0)	3,176 (n = 4)	11,253 (n = 6)	15,498 (n = 16)
Disease management and follow-up	827 (n = 4)	848 (n = 4)	13,668 (n = 9)	10,826 (n = 7)	26,169 (n = 24)
Education/training	10,051 (n = 6)	1,977 (n = 3)	15,937 (n = 13)	28,894 (n = 12)	56,859 (n = 34)
Risk factor reduction program	5,969 (n = 3)	0 (n = 0)	3,356 (n = 7)	11,337 (n = 7)	20,662 (n = 17)
Screening	8,907 (n = 5)	1,111 (n = 3)	33,544 (n = 11)	12,138 (n = 11)	55,700 (n = 30)
Treatment	51 (n = 1)	0 (n = 0)	3,158 (n = 3)	1,199 (n = 1)	4,408 (n = 5)
Other	0 (n = 0)	0 (n = 0)	607 (n = 3)	0 (n = 0)	607 (n = 3)

Abbreviations: CPD, chronic pulmonary disease; CVD, cardiovascular disease.

^a^ Values in columns may not sum to total value for n because a project could serve people in more than 1 category.

^b^ Cross-cutting refers to projects that address 2 or more chronic disease areas. Referrals were not reported as a separate strategy until fiscal year 2009–2010.

Awareness and media was a commonly used strategy. Because common metrics for broadcast media often include media impressions, several projects reported large reach numbers. For FY 2008–2009, 25 CCPD projects reported reaching 929,217 Coloradoans by using awareness/media strategies.

Reach can also be summarized for a specific disease and strategy, for example, the number of people screened specifically for hypertension. When reporting is implemented over several cycles, trends can be studied. Such descriptive summaries can help state staff assess the feasibility of demonstrating population-level impact through surveillance or other reporting systems. These data help staff understand whether interventions had sufficient saturation (or penetration) of populations to be detected using standard public health surveillance instruments (eg, Behavioral Risk Factor Surveillance System). Because each project described its target population, geographic region, and expected reach, reported reach data can be compared with the demographics provided for the given geographic area to approximate the number of people reached relative to a known denominator. Although these may be imperfect estimates, the combined reach of several projects using similar approaches could be sufficient to contemplate potential outcomes and more in-depth, focused evaluations.

### Assessing implementation across projects

Most implementation data are qualitative, and evaluation summaries are primarily narrative, such as this excerpted example based on the most widely used strategy, education and training:

How the education and training strategy was delivered: Education involved distributing information on specific chronic diseases or risk factors such as [cardiovascular disease], pulmonary conditions, diabetes, obesity, various types of cancer, poor nutrition, and physical inactivity. Most information was delivered through oral presentations or lectures, accompanied by written materials such as pamphlets or PowerPoint slides. Some participatory physical activity was also reported; examples are snowboarding, yoga, and dance.

More focused summaries are also possible (Table 3).

**Table 3:** Sample Summary of Reported Implementation Details From Cardiovascular Disease–Focused Screening Projects (N = 11)

Characteristic	Implementation Detail
Number of people screened	33,544
Who participated?	Adult community members, employees and spouses at worksites, women over age 40 with low income, individuals with low incomes and risk factors, residents in rural counties, adults and adolescents with type 1 diabetes mellitus
What types of screening?	BMI, lipids, blood pressure, HbA1c, glucose, Framingham risk assessment, physical activity, nutrition
How were screenings delivered?	Community-based computer kiosks, automated point-of-care lipid machine, primary care office visits, community health workers, public health nurses, walk-in health clinics
What happened after screening?	Education or counseling on health risks including high blood pressure, high cholesterol/lipids, HbA1c; referral to health care providers

Abbreviations: BMI, body mass index; HbA1c, hemoglobin A1c.

For some intervention delivery strategies, the reach and implementation (R&I) system can produce quantitative implementation data. We extracted data on education and training for indirect audiences to illustrate how many intermediaries received training or education. In FY 2009–2010, for 16 CCPD projects, 957 people among indirect audiences received education and training: 603 clinical providers/staff, 73 community health workers/health educators, 68 nonclinical program staff/executives, and 213 mixed audiences (combinations of the above). By looking at similar projects, we were able to identify opportunities for standardization of implementation and data collection. For example, several projects focused specifically on screening target populations for heart disease risks: high blood pressure, high cholesterol, and elevated body mass index. Implementation data highlighted opportunities to standardize screening services and data, realizing efficiencies in program delivery and the potential for better participant outcomes. This activity is separate from contract monitoring, in which program staff who know the details of the projects can apply their own judgment about how well individual projects are meeting their proposed objectives.

### Monitoring and planning

DOH monitors compare implementation data with contractual work plans and invoices for reimbursement to determine whether the state is receiving what it contracted for and identifying opportunities for technical assistance. DOH content experts in specific diseases or strategies complemented the contract monitoring function by reviewing whether implementation data aligns with guidelines and expectations. For example, a report of a positive screening outcome should trigger reporting of referral or treatment of the identified condition. If no follow-up is reported, technical assistance can be initiated. More concretely, several projects used WISEWOMAN-like approaches (16) to address cardiovascular disease among underserved women. Content experts assessed whether project implementation occurred with fidelity, focusing on whether screenings included at least 2 blood pressure readings, in line with the evidence. If not, staff worked with the project staff to improve the implementation of the project to conform to “best practices.”

Reach and implementation data also enable DOH staff and oversight committees to review the populations served and examine the portfolio of strategies and diseases in summary and in detail. Regular reports to the oversight bodies (grant review committees and Board of Health) provided information on the diverse strategies across disease categories and FYs to broadly assess whether the portfolio of projects aligned with funding objectives.

## Lessons From the Reporting System Implementation and Results

Development of a uniform reporting system can yield useful information to support monitoring and strategic review of complex portfolios of public health projects. Through Colorado’s R&I system, projects consistently reported standardized data about diverse program recipients using common demographic categories and reporting terminology. Implementation processes have become more transparent and evaluable as a result. Considerable time and effort must be devoted to system planning and development. The Colorado R&I system development took approximately 12 months from initial engagement of the evaluation team to debugged system operation.

Quarterly reviews of individual project reports proved essential for improving data quality but were labor-intensive during the first 3 quarters when feedback to reporting entities was essential to achieve complete and reliable data at the end of the FY. Quarterly reviews were typically quick and problems were corrected with a brief e-mail or telephone call (less than 30 minutes per year); for a handful of projects, intensive technical assistance was needed (30 to 90 minutes per year). Reporting system developers should plan sufficient staffing to address ongoing training and technical assistance needs. Furthermore, grant monitors at the DOH could add efficiency by having a more active role during the quarterly data quality reviews.

The R&I reporting system supports multiple levels of implementation review, from specific projects in detail to all projects in broad-brush summary. For these analytic levels to be meaningful, projects must be based on well-evaluated practices, a criterion that should be made clear to grant application reviewers before projects are considered for funding. DOH staff must also be sufficiently trained and informed to work with an evaluation paradigm that directly measures implementation while relying mainly on previous research as evidence of likely effectiveness.

The R&I reporting system is not designed for evaluating outcomes at the individual project level; such evaluations are prohibitively costly in aggregate when a program funds scores of grantees. The system can assist such evaluations in conjunction with separately measured outcome data by providing the necessary data about intervention delivery.

Public health programs that address multiple diseases, employ varied strategies, or serve diverse populations will generate varied implementation data. The R&I system supports only broad integration and summary of these data. Further elaboration of the reporting forms would allow closer analysis of implementation processes among more homogeneous groups of projects. Proven public health programs or methods often must be adapted to be widely disseminated. Details about the adaptation process can indicate the feasibility and fidelity of intervention implementation among diverse populations (17,18). This adaptation requires stakeholder establishment of evidence-based performance standards and criteria (9) but would improve assessment of fidelity to protocols and the value of a system that depends on predetermination of intervention method effectiveness. Finally, in the absence of clearly established denominators, reach data from the R&I system are unlikely to deliver data sufficient for estimating effects on populations. However, where funding for full-scale, project-level evaluations is limited, the data are a starting point to assess a portfolio of projects. This assessment must be taken in light of the overall goals of the program (“Is this the right mix of projects for our goals?”) balanced with the scope, objectives, funding, and methods of individual projects (“Is this project achieving its own objectives?”).

## Conclusions

A simple, form-based reporting system has been developed and successfully used to provide evaluation components in public health initiatives that involve multiple and diverse projects, organizations, sites, and populations. A meaningful set of common data elements can be collected using widely available spreadsheet software with dispersed (noncentralized) data entry. The system can easily be refined to support evaluation studies of public health strategies that depend on translation of evidence-based methods to reduce risky behaviors and promote healthy behaviors among large populations.
